# Podoplanin promotes the carcinogenicity of gastric cancer by activating ezrin and mediating the crosstalk between tumour cells and cancer‐associated fibroblasts

**DOI:** 10.1113/EP090172

**Published:** 2023-03-28

**Authors:** Yueli Tian, Xin Chen, Xiaodong Wang, Ying Song

**Affiliations:** ^1^ Gastroenteric Medicine and Digestive Endoscopy Center The Second Hospital of Jilin University Changchun Jilin China

**Keywords:** CAFs, ezrin, gastric cancer, PDPN, tumourigenesis

## Abstract

Gastric cancer (GC) is a frequent malignant disease and the main cause of cancer‐related death in the world. Podoplanin (PDPN) has been proved to be involved in the progression of various cancers. However, the role and biological mechanism of PDPN in GC are still vague. In our study, we detected the expression of PDPN in GC tissues and cell lines using RT‐qPCR, western blot and datasets. The overall survival of GC patients was analysed with a Kaplan–Meier plot. The effects of PDPN overexpression and silencing on GC cell progression were assessed by Cell Counting Kit‐8, flow cytometry and a wound healing assay. Besides, the modulation of PDPN on ezrin activation was investigated. We further explored the role of PDPN in the crosstalk between GC cells and cancer associated fibroblasts (CAFs). Results showed that PDPN was upregulated in GC tissues and cell lines. High expression of PDPN was correlated with poor prognosis of GC patients. PDPN positively regulated the viability, migration and invasion, but inhibited apoptosis, of GC cells by mediating the activation of ezrin. Meanwhile, the change in PDPN in GC cells activated CAFs and promoted the production of cytokines secreted by CAFs, which induced the progression of GC cells. These findings may provide a novel target for GC therapy.

## Abstract (highlights)

- **What is the central question of this study?**
  To reveal the role and biological mechanism of PDPN in the progression of gastric cancer.
- **What is the main finding and its importance?**
  This study focused on a prognostic predictor, PDPN, which acted as a promoter in the progression of gastric cancer through the activation of Ezrin expression and CAFs. This finding may expand a new route for the gene‐targeted therapy in gastric cancer.

## 1 INTRODUCTION

Gastric cancer (GC), a common malignant disease, has become one of the main causes of cancer‐related death globally. Since most patients with GC are diagnosed at an advanced stage, treatment options are limited and the 5‐year survival rate has declined for most patients (Qiao et al., 2019). Researchers have demonstrated the involvement of various prognostic markers in regulating the progression of GC. However, the underlying mechanisms of GC initiation and development are still vague, and exploring novel therapeutic targets may contribute to improving the survival rate and quality of life of GC patients.

Podoplanin (PDPN), a mucin‐type transmembrane protein, is conserved in different species (Astarita et al., 2012). Reportedly, PDPN is not only expressed in various normal cells like type I alveolar epithelial cell (Rishi et al., 1995), lymphatic endothelium and kidney podocytes (Koop et al., 2008; Wetterwald et al., 1996), but also frequently upregulated in numerous malignant tumours, including hypopharyngeal cancer (Wang et al., 2017) and squamous cell carcinoma (Li & Tang, 2021; Schwab et al., 2021), as well as in tumour stroma‐containing cancer‐associated fibroblasts (CAFs) (Jsa et al., 2020). Therefore, PDPN performs an active role in organ development, cell movement and oncogenesis. In particular, PDPN is involved in tumour cell growth, invasion, migration, metastasis and inflammation (Krishnan et al., 2018). For example, PDPN promoted tumour cell metastasis through activating platelet C‐type lectin‐like receptor 2 via induction of tumour cell‐induced platelet aggregation (Chang et al., 2015). The upregulation of PDPN was associated with higher probability of venous thrombosis in an animal model of ovarian cancer (Sasano et al., 2022). Besides, the interaction between cancer cells and surrounding CAFs in the tumour microenvironment (TME) plays an important role in cancer progression (Ji et al., 2021). Interestingly, PDPN also has been proved to participate in the regulation of the TME (Sakai et al., 2018). PDPN expressed in CAFs contributes to overall tumour growth due to TME‐mediated angiogenesis and immunosuppression. There was an enhanced drug‐resistant effect of lung adenocarcinoma cells observed in PDPN‐positive CAFs (Yoshida et al., 2015). In addition, the enhanced PDPN expression in CAFs was confirmed to accelerate the invasion of lung adenocarcinoma cells by promoting epithelial–mesenchymal transition (EMT) (Naito et al., 2016) and induce an immunosuppressive microenvironment through increasing transforming growth factor‐β and interleukin (IL)‐10 to escape the immune response of the host (Suzuki et al., 2021). Also, increased ezrin in the presence of PDPN‐positive CAFs facilitated aggressiveness in lung adenocarcinoma (Suzuki et al., 2015). Furthermore, biological agents targeting PDPN, including antisera and CAR‐T cells, have been shown to suppress cancer development in preclinical studies (Krishnan et al., 2018). However, the exact role and mechanism of PDPN in GC remain unclear.

In our study, we evaluated the expression of PDPN in GC tissues and investigated the biological function and regulatory mechanism of PDPN in GC progression. In addition, we explored the effect of PDPN on the interaction between GC cells and CAFs in TME. This study represents one step to our goal of providing an effective therapy for patients with GC.

## 2 METHODS

### 2.1 Tissue specimens

A total of 35 pairs of primary GC tissues and adjacent non‐cancer tissues more than 5 cm away from the tumour border were obtained from patients with GC undergoing resection surgery at the Second Hospital of Jilin University from 2017 to 2020. None of them had undergone any antitumour therapy before surgery. Clinical information for the GC patients is shown in Table 1.

**TABLE 1:** Relevant clinicopathological characteristics of GC patients and PDPN expression.

		PDPN expression		
Clinical or pathological characteristic	Cases	High (*n* = 20)	Low (*n* = 15)	*P*
Age (*n*)				0.803
<65 years	14	7	7	
≥65 years	21	13	8	
Sex (*n*)				0.714
Female	16	10	6	
Male	19	10	9	
Pathological stage (*n*)				0.335
Ⅰ–Ⅱ	22	12	10	
III–IV	13	8	5	
T classification (*n*)				0.0327
T1∼T2	13	4	9	
T3∼T4	22	16	6	
Distant metastasis (*n*)				0.0164
Positive	20	17	3	
Negative	15	3	12	
Lymph node metastasis (*n*)				0.0271
Yes	19	15	4	
No	16	5	11	

### 2.2 Ethical approval

The performance of our research and the use of cell materials in this study were approved by the Ethics Committee of the Second Hospital of Jilin University (Approval Number: SY201906011) and was in accord with the *Declaration of Helsinki*, apart from registration in a database. There was prior written informed consents from the subjects.

### 2.3 Survival analysis

For analysis of overall survival of 35 surgical cancer subjects, GraphPad Prism 8.0 (GraphPad Software Inc., San Diego, CA, USA) was employed to map the survival curve. For analysis of overall survival of online GC samples (Access dataset number: 208233‐at), Kaplan–Meier univariate survival analysis was conducted using a Kaplan–Meier plot (http://kmplot.com/analysis/index.php?p=service&cancer=gastric). The time from beginning of surgery to death from any cause or the last date of follow‐up was defined as overall survival. The median value of PDPN expression was selected as the critical value to classify the subjects into high‐expression and low‐expression groups. The hazard ratio with 95% confidence intervals was calculated.

### 2.4 Cell culture

The human GC cancer cell lines (AGS, HGC‐27, MKN‐45, SNU‐1 and Hs.746T) and human gastric mucosal cell line GES‐1 were purchased from American Type Culture Collection (ATCC, Manassas, VA, USA) and maintained in RPMI‐1640 medium (Sigma‐Aldrich, St Louis, MO, USA) with 10% fetal bovine serum (FBS; Thermo Fisher Scientific, Waltham, MA, USA) in 5% CO_2_ at 37°C.

Human CAFs were isolated and obtained from fresh samples of GC tissues according to differential trypsinization with a modified protocol (Grunberg et al., 2021; Ma et al., 2018). Primary CAFs derived from the patients with GC were confirmed by detection of CAF markers (CD90 positive and CD45 negative) and maintained in Dulbecco's modified Eagle's medium/F12 (Sigma) containing 10% FBS and 1% penicillin–streptomycin in 5% CO_2_ at 37°C.

CAFs were co‐cultured with the SNU‐1 cell line. An indirect co‐culture system was performed using a six‐well Transwell culture system (Corning Incroporated, NY, USA). Isolated CAFs (2 × 10^5^ cells per well) were cultured in the lower chambers, the SNU‐1 cell line (5 × 10^5^ cells per well) was seeded in the upper chambers. After incubation for 72 h, the cells were used in functional assays.

### 2.5 Cell transfection

Small interfering RNAs (siRNAs) targeting PDPN and overexpression plasmid of PDPN and Rho‐associated protein kinase (ROCK) were designed and synthesized by GenePharma (Shanghai, China). SNU‐1 and AGS cells were seeded in a 24‐well plate (5 × 10^4^ per well). For knockdown analysis, we transfected SNU‐1 cells with 50 nM PDPN siRNAs along with 5 μg ROCK overexpression plasmid for 48 h; negative control (NC) siRNA (50 nM) was used as control. AGS cells were transfected with PDPN overexpression plasmids (PDPN vector; 1, 2 and 5 μg) and pcDNA3.1 empty vector (5 μg; control vector; cat. no. V79020; Thermo Fisher Scientific) for 48 h. The transfection process was carried out with the use of Lipofectamine 3000 transfection reagent (cat. no. 18324010; Thermo Fisher Scientific). After transfection, cells were used for functional assays.

### 2.6 Cell treatment

After transfection, AGS cells were exposed to 10 μM ezrin phosphorylation inhibitor NSC668394 (cat. no. 341216; Sigma) for 24 h. Besides, the SNU‐1 and CAFs co‐cultured system was treated with 40 μg exogenous recombination PDPN (rPDPN) (cat. no. 81154‐R02H; Sino Biological, Beijing, China) at the 24‐h co‐culture period.

### 2.7 Cell counting kit assay

GC cells were seeded in 96‐well plates (5 × 10^3^ cells per well). After incubation for 24, 48, 72 and 96 h, 10 μl Cell Counting Kit (CCK‐8) solution from the CCK‐8 Kit (cat. no. C0041; Beyotime, Shanghai, China) was added to each well and incubated at 37°C for 4 h. The optical density at 450 nm in each well was measured using a microplate reader (Bio‐Rad Laboratories, Hercules, CA, USA) for cell viability. The final presentation of cell viability at different time points was shown by normalizing OD value to the 24 h time point of the control group for the clearer exhibition of changes.

### 2.8 Cell apoptosis assay

After the transfection for 48 h, the cells (1 × 10^5^ cells per well) were collected and re‐suspended with phosphate‐buffered saline. Then, 5 μl of annexin V–fluorescein isothiocyanate and propidium iodide (cat. no. 331200; Thermo Fisher Scientific) were added to the cells and they were incubated in the dark at 25°C for 15 min. After resuspension, apoptotic cells were detected using a flow cytometer (BD Biosciences, San Jose, CA, USA).

### 2.9 Wound healing assay

Cells were inoculated into six‐well culture plates (1 × 10^6^/well). They were then transfected according to the demand of this assay and cultured in a 37°C, 5% CO_2_ in an incubator until 90% confluence. After starvation in the serum‐free medium for 12 h, scratches were made along the cells using a sterile 200 μl pipette tips. Next, the cell debris generated by the scratch was washed away. The cells were photographed at 0 and 24 h after wound scratching in three random visual fields using an optical microscope (Leica, Wetzlar, Germany; magnification: ×100). The cell migration distance was calculated using Image Pro‐Plus 6.0 software (Media Cybernetics, Rockville, MD, USA).

### 2.10 Transwell assay

A 24‐well Corning Transwell (Corning Incroporated, NY, USA) chamber (upper) was pre‐coated with Matrigel (cat. no. 356234; BD Biosciences) for invasion assay. The density of GC cells was 2 × 10^4^ per chamber and the cells were seeded into the upper chamber with serum‐free medium. FBS (10%) was prepared in the bottom chamber. The incubation lasted for 24 h. Then, the invasive cells attached to the lower surface were peeled and treated with methanol (4%, 0.5 h) and crystal violet (0.1%, 15 min). The final data were calculated based on three fields randomly selected under an optical microscope (Leica; magnification: ×100).

### 2.11 RT‐qPCR

Total RNA was isolated from tissues or cells using TRIzol reagent (cat. no. 15596018; Thermo Fisher Scientific) and reverse‐transcribed by PrimeScript RT Reagent Kit (cat. no. RR037Q; Takara, Shiga, Japan). The RT‐qPCR was performed using QuantiTect SYBR Green PCR Kits (cat. no. 204143; Qiagen, Hilden, Germany), specific primers and a real‐time PCR system (cat. no. 4376600; StepOnePlus, Thermo Fisher Scientific). The gene mRNA expression was calculated using the $2^{-\Delta \Delta C_{t}}$ method and normalized against glyceraldehyde 3‐phosphate dehydrogenase (GAPDH) gene expression. The specific primers are as followed: PDPN forward: 5′‐TTCATTGGTGCAATCATCGT‐3′, reverse: 5′‐AGAGGAGCCAAGTCTGGTGA ‐3′; vascular endothelial growth factor A (VEGFA) forward: 5′‐ATCGAGTACATCTTCAAGCCAT‐3′, reverse: 5′‐GTGAGGTTTGATCCGCATAATC‐3′; GAPDH forward: 5′‐GCACCGTCAAGGCTGAGAAC‐3′, reverse: 5′‐TGGTGAAGACGCCAGTGGA‐3′.

### 2.12 Western blotting

Tissues and cells were lysed in RIPA buffer (cat. no. R0020; Solarbio Science and Technology, Beijing, China) to recover total protein. Then the protein quantification was measured using a bicinchoninic acid assay kit (cat. no. 23227; Thermo Fisher Scientific). Twenty micrograms of protein was loaded onto SDS‐PAGE and transferred to polyvinylidene difluoride membranes (cat. no. 1620177; Bio‐Rad). After blocking, protein levels were detected by specific primary antibodies from Abcam (Cambridge, MA, USA) against PDPN (ab236529, 1:1000), ezrin (ab4069, 1:500), Ki67 (ab16667, 1:1000), E‐cadherin (ab40772, 1:10000), N‐cadherin (ab76011, 1:1000), cleaved‐caspase 3 (ab2302, 1:5000), α‐smooth muscle actin (α‐SMA; ab5831, 1:1000), fibroblast activation protein (FAP; ab207178, 1:1000), fibroblast‐specific protein 1 (FSP‐1; ab124805, 1:1000) and GAPDH (ab8245, 1:2500), followed by the corresponding horseradish peroxidase‐conjugated secondary antibodies (ab6721/ab205719, 1:5000) and visualization. GAPDH was used as the internal control.

### 2.13 Enzyme linked immunosorbent assay

Enzyme linked immunosorbent assays (ELISA) were performed using the collected supernatant of the CAF co‐culture system according to the manufacturer's instructions. The ELISA kits were purchased from CUSABIO (Wuhan, China; cat. nos CSB‐E04638h, CSB‐E04641h and CSB‐EQ004783HU) and used to determine the production of IL‐6, IL‐8 and CCL2 using a microplate reader (Bio‐Rad) at a wavelength of 450 nm.

### 2.14 Statistical analysis

The results are presented as means ± standard deviation (SD) from three independent experiments with triple replicates per experiment. The statistical difference comparisons between two or multiple different experimental groups were performed using Student's *t*‐test or one‐way analysis of variance (ANOVA) with Prism software (Version 8.0). *P < 0.05 and **P < 0.01 were indicated as a significant statistical difference.

## 3 RESULTS

### 3.1 PDPN was highly expressed in GC and associated with poor prognosis

**FIGURE 1:**
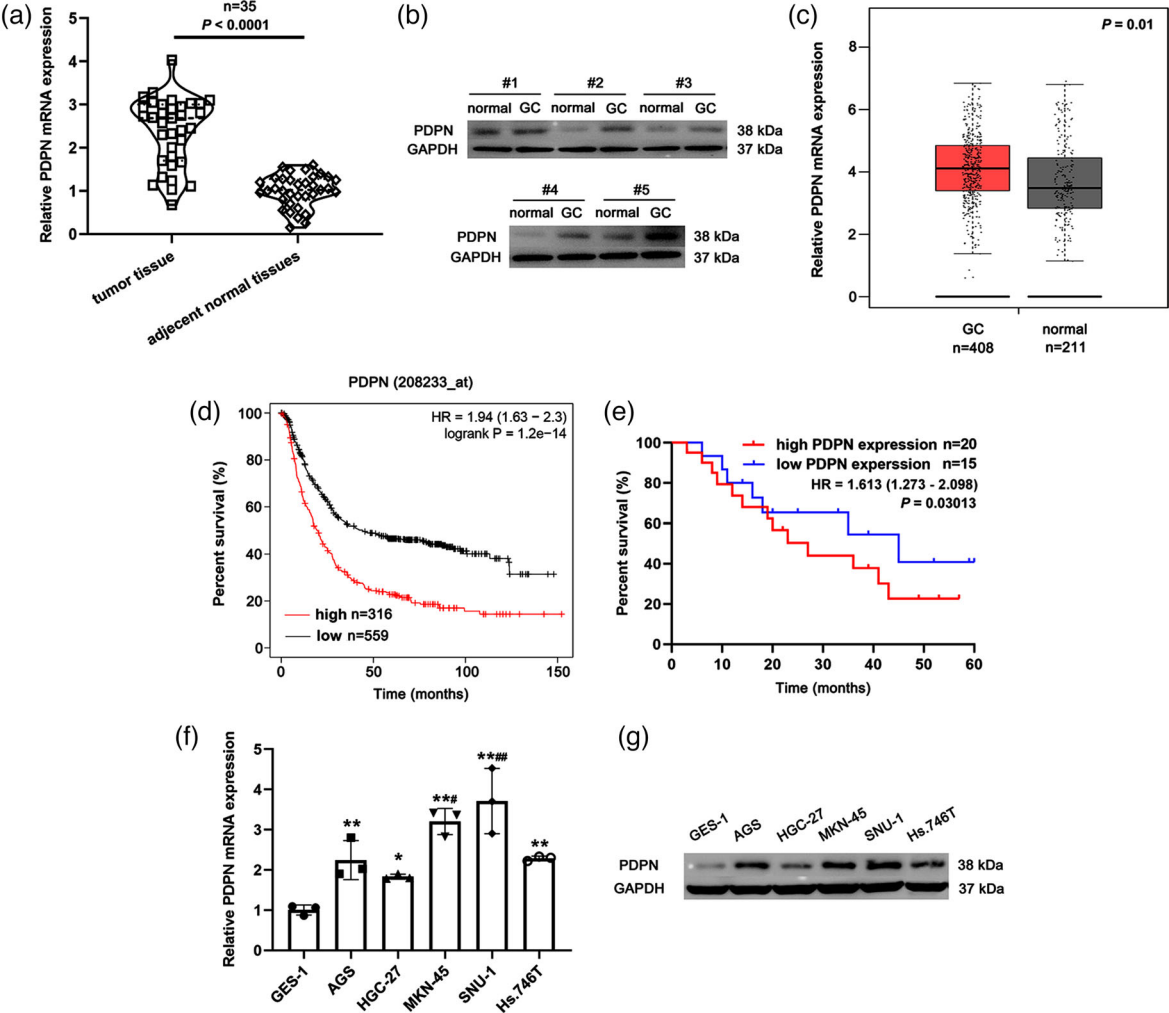
PDPN was highly expressed in GC samples and associated with poor prognosis of GC patients. (a) The relative mRNA expression of PDPN in paired tumour tissues and adjacent normal tissues (*n* = 35). ^***^ *P* < 0.001 versus adjacent tumour tissues. (b) The protein expression of PDPN in five pairs of tumour tissues and adjacent non‐tumour tissues. (c) The relative mRNA expression of PDPN from TCGA database (GEPIA) in GC tissues (*n* = 408) and normal tissues (*n* = 201). ^***^ *P* < 0.001 versus GC group. (d) Survival curve based on high PDPN expression (*n* = 316) and low PDPN expression (*n* = 559) from a Kaplan–Meier plot of survival analysis platforms (access dataset number: 208233‐at). (e) High PDPN expression (*n* = 20) was associated with worse overall survival of GC patients. *P =* 0.03013 versus low PDPN expression group (*n* = 15). (f) The relative mRNA expression of PDPN in GC cell lines. ^*^ *P* < 0.05 and ^**^ *P* < 0.01 versus GES‐1 cell line; ^#^ *P* < 0.05 and ^##^ *P* < 0.01 versus AGS cell line. (g) The protein expression of PDPN in GC cell lines. All data are presented as means ± SD (*n* = 3). HR, hazard ratio.

We first analysed the expression of PDPN in GC tissues. The results showed that PDPN was highly expressed in GC tissues compared with adjacent normal tissues (*P* < 0.0001, Figure 1a,b). The increased expression of PDPN was also found in GC tissues based on the TCGA database using GEPIA2 online software (http://gepia2.cancer‐pku.cn/#index) (*P* = 0.01, Figure 1c). Patients with high expression of PDPN showed a poor survival outcome, both in the populations of our study (*P* = 1.2 × 10^−14^, Figure 1d) and in dataset 208233‐at (*P* = 0.0301, Figure 1e). Consistently, GC cell lines exhibited increased mRNA and protein expression of PDPN compared with the GES‐1 cell line, which was used as the control in this cellular study (AGS, *P* = 0.00651; HGC‐27, *P* = 0.0136; MKN‐45, *P* = 0.00361; SNU‐1, *P* = 0.00821; Hs.746T, *P* = 0.00228; Figure 1f,g).

### 3.2 PDPN mediated GC cell viability, apoptosis, invasion and migration

**FIGURE 2:**
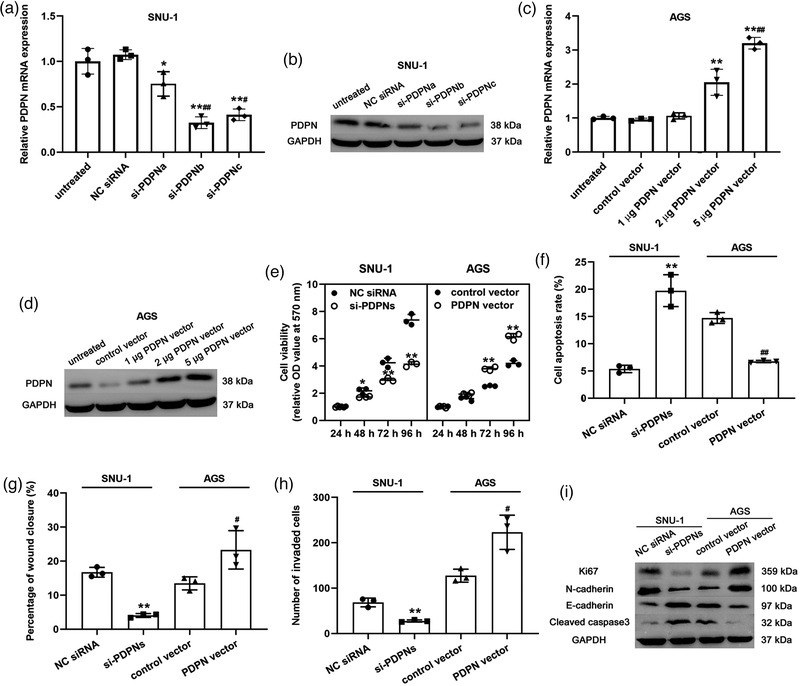
PDPN mediated GC cell viability, apoptosis, invasion and migration. (a, b) The relative mRNA (a) and protein (b) expression of PDPN after transfection with si‐PDPNs. ^*^ *P* < 0.05 and ^**^ *P* < 0.01 versus untreated group, ^#^ *P* < 0.05 and ^##^ *P* < 0.01 versus si‐PDPNa group. (c, d) The relative mRNA (c) and protein (d) expression of PDPN after transfection with PDPN vector. ^**^ *P* < 0.01 versus untreated group; ^##^ *P* < 0.01 versus 2 μg PDPN vector group. (e) The cell viability of GC cells. ^**^ *P* < 0.01 versus NC siRNA or control vector group in SNU‐1 and AGS cells, respectively. (f) The apoptosis rate of GC cells. ^**^ *P* < 0.01 versus NC siRNA group; ^##^ *P* < 0.01 versus control vector group. (g, h) The percentage of wound closure (g) and number of migrated cells (h) of GC cells. ^**^ *P* < 0.01 versus NC siRNA group; ^#^ *P* < 0.05 versus control vector group. (i) The protein expression of Ki67, E‐cadherin, N‐cadherin and cleaved‐caspase 3. All data are presented as means ± SD (*n* = 3).

To assess the role of PDPN in GC progression, loss‐of‐function and gain‐of‐function assays were conducted in vitro. The transfection of PDPN siRNAs significantly inhibited the expression of PDPN in SNU‐1 cells (NC siRNA, *P* = 0.451; siPDPNa, *P* = 0.0236; siPDPNb *P* = 0.00682; siPDPNc *P* = 0.00703; Figure 2a,b), while the PDPN expression in AGS cells was induced after the transfection of PDPN vector (Control vector, *P* = 0.531; 1 μg PDPN vector, *P* = 0.433; 2 μg PDPN vector, *P* = 0.0261; 5 μg PDPN vector, *P* = 0.0137; Figure 2c,d). Based on the results, the most effective treatments of PDPN siRNA‐mix (a combination of si‐PDPNb and si‐PDPNc) and PDPN vector (5 μg) were used for subsequent experiments. Furthermore, the CCK‐8 assay results implied that PDPN knockdown suppressed SNU‐1 cell viability, while PDPN overexpression promoted AGS cell viability (SNU‐1: 24 h, *P* = 0.712; SNU‐1: 48 h, *P* = 0.0395; 72 h, *P* = 0.00815; 96 h, *P* = 0.00519; AGS: 24 h, *P* = 0.661; AGS: 48 h, *P* = 0.193; 72 h, *P* = 0.00694; 96 h, *P* = 0.00138; Figure 2e). Meanwhile, PDPN silencing induced an increased rate of apoptotic cells (SNU‐1: si‐PDPNs, *P* = 0.00837; AGS: PDPN vector, *P* = 0.00604; Figure 2f) and reduced the migration (SNU‐1: si‐PDPNs, *P* = 0.00573; AGS: PDPN vector, *P* = 0.0328; Figure 2g) and invasion (SNU‐1: si‐PDPNs, *P* = 0.00731; AGS: PDPN vector, *P* = 0.0173; Figure 2h) of GC cell lines. A previous study indicated that PDPN acted as an essential participant in the process of EMT in oesophagus squamous carcinoma (Watanabe et al., 2020). Reduced E‐cadherin expression and elevated N‐cadherin expression are hallmarks of EMT and are associated with an increased risk of cancer metastasis (Loh et al., 2019). The western blot results showed that the expression of Ki67 and N‐cadherin was decreased and the expression of E‐cadherin and cleaved‐caspase 3 was increased by transfecting with si‐PDPNs (Figure 2i). Nevertheless, the transfection of PDPN vector exhibited opposite effects in AGS cells (Figure 2f–i).

### 3.3 PDPN promoted the progression of GC by activating ezrin

**FIGURE 3:**
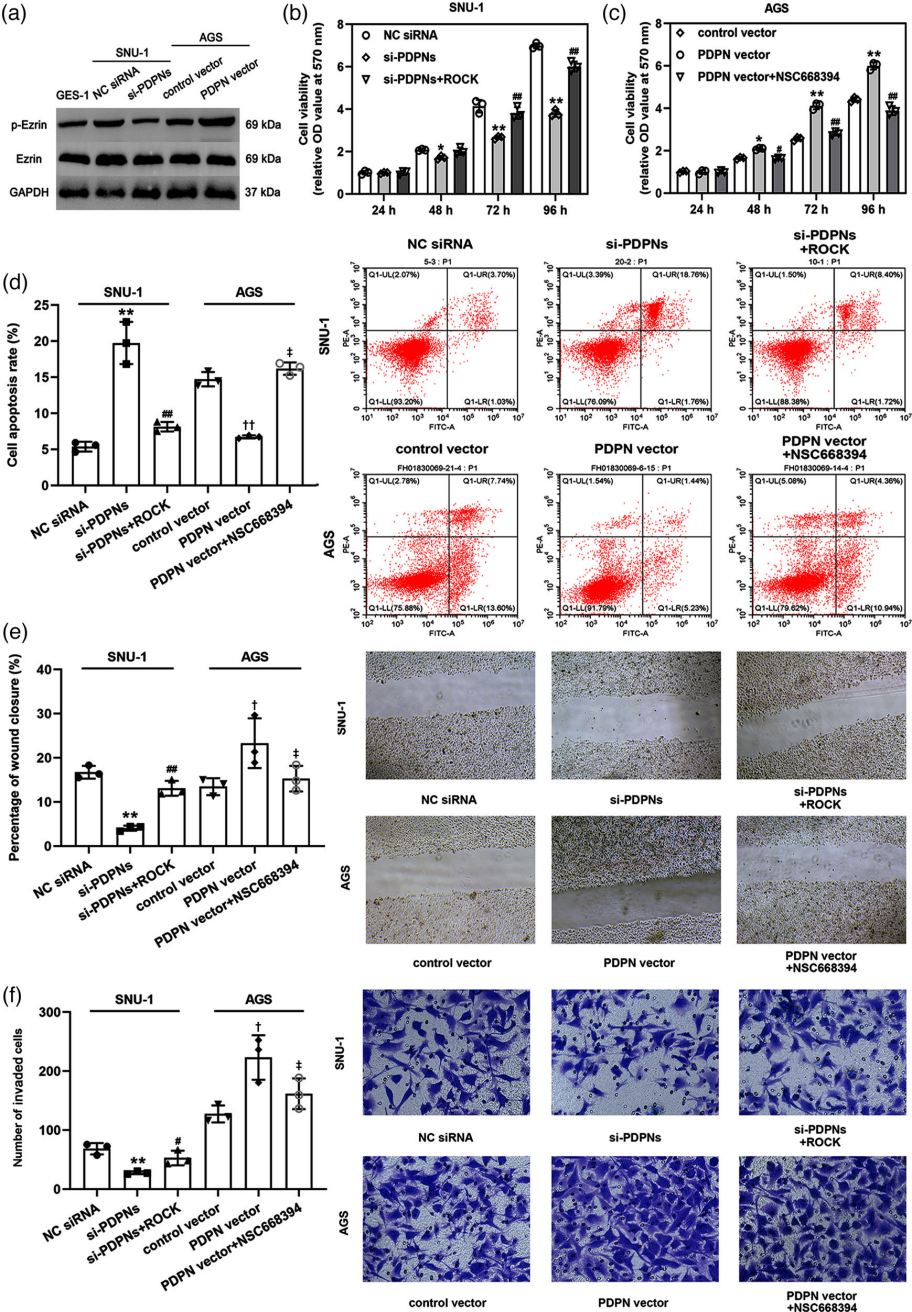
PDPN promoted the progression of GC via activating ezrin. (a) The protein expression of ezrin in cultured cells. (b and c) The cell viabilities of SNU‐1 and AGS cells with or without the treatment of ROCK. ^*^ *P* < 0.05 and ^**^ *P* < 0.01 versus NC siRNA group; ^##^ *P* < 0.01 versus si‐PDPNs group. (c) The cell viability of AGS cells with or without the treatment of NSC668394. ^*^ *P* < 0.05 and ^**^ *P* < 0.01 versus control vector; ^#^ *P* < 0.05 and ^##^ *P* < 0.01 versus PDPN vector group. (d–f) The apoptosis rate (d), percentage of wound closure (e), and number of migrated cells (f) of SNU‐1 and AGS cells. ^**^ *P* < 0.01 versus NC siRNA group; ^#^ *P* < 0.05 and ^##^ *P* < 0.01 versus si‐PDPNs group; ^†^ *P* < 0.05 and ^††^ *P* < 0.01 versus control vector; ^‡^ *P* < 0.05 versus PDPN vector group. All data are presented as mean ± SD (*n* = 3).

Previous studies suggested that PDPN regulated EMT by mediating the expression of ezrin (Krishnan et al., 2013; Sikorska et al., 2019). Thus, we assessed the role of ezrin in PDPN‐mediated GC progression. As shown in Figure 3a, ezrin was activated in SNU‐1 and AGS cells compared with GES‐1 cells. Compared with the NC siRNA and control vector groups, PDPN knockdown reduced phosphorylation of ezrin in SNU‐1 cells and PDPN overexpression led to an increased phosphorylation of ezrin in AGS cells. To promote ezrin signalling, rho‐associated protein kinase (ROCK) was overexpressed by transfecting ROCK vector (Figure S1). Subsequently, we evaluated the role of ezrin activation during GC progression. As presented in Figure S2, ROCK overexpression‐induced ezrin activation dramatically promoted cell viability (24 h, *P* = 0.372; 48 h, *P* = 0.0471; 72 h, *P* = 0.00596; 96 h, *P* = 0.00708; Figure S2A), migration (*P* = 0.00815; Figure S2D) and invasion (*P* = 0.00751; Figure S2E) and inhibited cell apoptosis (*P* = 0.0302; Figure S2C); however, the depression of ezrin induced by NSC668394 showed the opposite effect on cell viability (24 h, *P* = 0.637; 48 h, *P* = 0.00861; 72 h, *P* = 0.00705; 96 h, *P* = 0.00690; Figure S2B), apoptosis (*P* = 0.00608; Figure S2C), migration (*P* = 0.0318; Figure S2D) and invasion (*P* = 0.00894; Figure S2E) of GC cells. The overexpression of ROCK, an activator of ezrin phosphorylation (Yin et al., 2018), attenuated the effects of PDPN silencing on ezrin activation (Figure S3), viability (SNU‐1‐si‐PDPNs: 24 h, *P* = 0.823; 48 h, *P* = 0.0337; 72 h, *P* = 0.00258; 96 h, *P* = 0.00129; SNU‐1‐si‐PDPNs+ROCK: 24 h, *P* = 0.964; 48 h, *P* = 0.0931; 72 h, *P* = 0.00635; 96 h, *P* = 0.00594; Figure 3b), apoptosis (SNU‐1‐si‐PDPNs, *P* = 0.00815; SNU‐1‐si‐PDPNs+ROCK, *P* = 0.00631; Figure 3d), invasion (SNU‐1‐si‐PDPNs, *P* = 0.0364; SNU‐1‐si‐PDPNs+ROCK, *P* = 0.0173; Figure 3e) and migration of GC cells (SNU‐1‐si‐PDPNs, *P* = 0.00362; SNU‐1‐si‐PDPNs+ROCK, *P* = 0.00518; Figure 3f). In addition, the effects of PDPN overexpression on the activation of ezrin (data not shown) and the biological functions of AGS cell line were reversed by ezrin inhibitor (NSC668394) (Lipreri da Silva et al., 2021) (AGS‐PDPN vector: 24 h, *P* = 0.863; 48 h, *P* = 0.0435; 72 h, *P* = 0.00863; 96 h, *P* = 0.00571; AGS‐PDPN vector+NSC668394: 24 h, *P* = 0.762; 48 h, *P* = 0.0433; 72 h, *P* = 0.00912; 96 h, *P* = 0.00662; Figure 3c. AGS‐PDPN vector, *P* = 0.00791; AGS‐PDPN vector+NSC668394, *P* = 0.00132; Figure 3d. AGS‐PDPN vector, *P* = 0.0196; AGS‐PDPN vector+NSC668394, *P* = 0.0381; Figure 3e. AGS‐PDPN vector, *P* = 0.0261; AGS‐PDPN vector+NSC668394, *P* = 0.0165; Figure 3f).

### 3.4 PDPN participated in CAF‐aggravated cell processes of SNU‐1 cells

**FIGURE 4:**
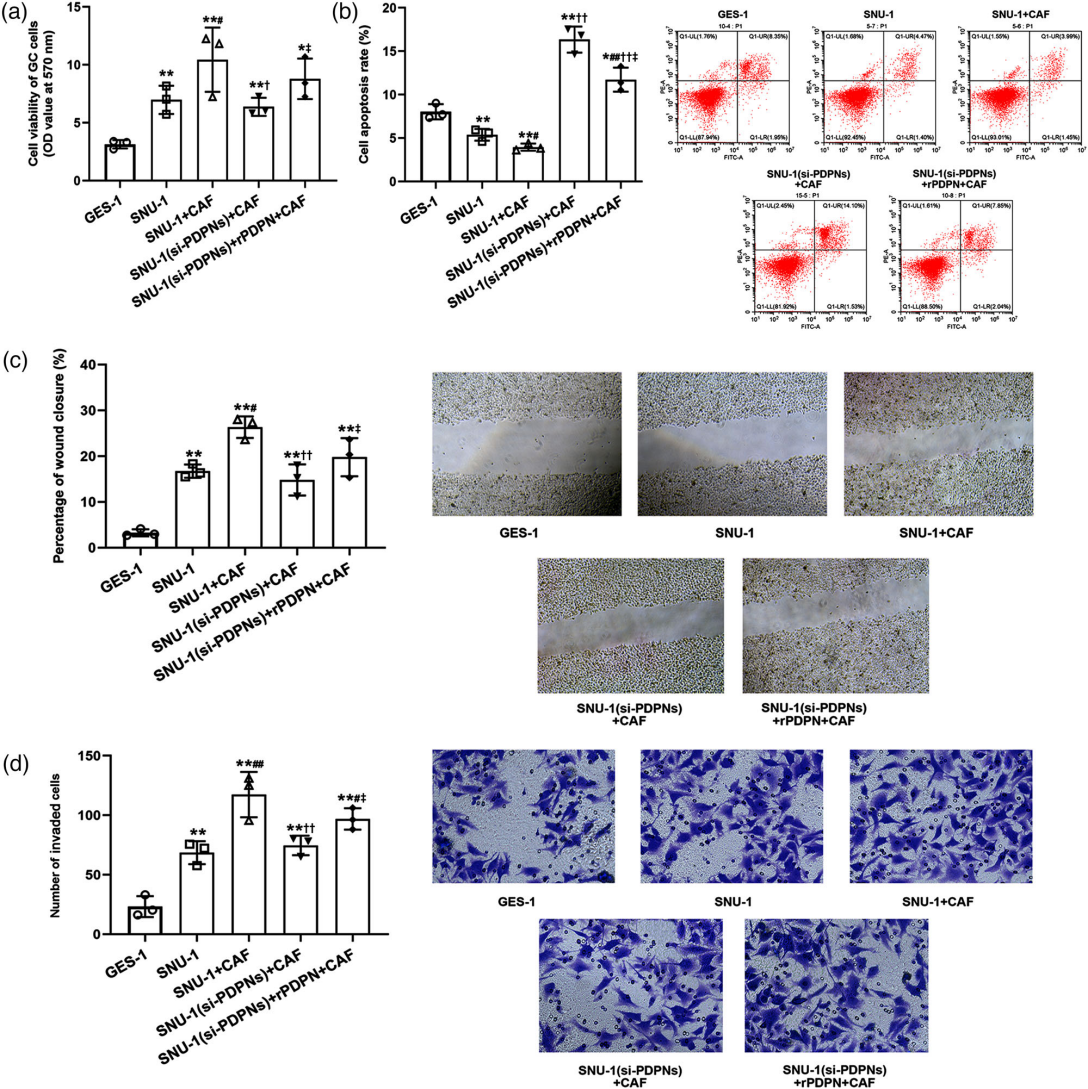
CAF motivated cellular processes of SNU‐1 cell via PDPN. (a) The cell viability of SNU‐1 cells after co‐culture with CAF cells. ^*^ *P* < 0.05 and ^**^ *P* < 0.01 versus GES‐1; ^#^ *P* < 0.05 versus SNU‐1; ^†^ *P* < 0.05 and ^†^^†^ *P* < 0.01 versus SNU‐1+CAF; ^‡^ *P* < 0.05 versus SNU‐1(si‐PDPNs)+CAF. (b) The cell apoptosis rate of SNU‐1 cells after co‐culture with CAF cells. ^*^ *P* < 0.05 and ^**^ *P* < 0.01 versus GES‐1; ^#^ *P* < 0.05 and ^##^ *P* < 0.05 versus SNU‐1; ^††^ *P* < 0.01 versus SNU‐1+CAF; ^‡^ *P* < 0.05 versus SNU‐1(si‐PDPNs)+CAF. (c, d) The percentage of wound closure (c) and number of migrated cells (d) in SNU‐1 cells after co‐culture with CAF cells. ^**^ *P* < 0.01 versus GES‐1 cell line; ^#^ *P* < 0.05 and ^##^ *P* < 0.01 versus SNU‐1 cell line; ^††^ *P* < 0.01 versus SNU‐1+CAF group; ^‡^ *P* < 0.05 versus SNU‐1 (si‐PDPNs) +CAF group. All data are presented as means ± SD (*n* = 3). The magnification of the image was 200x.

Subsequently, we further investigated the role of PDPN in CAF‐mediated alteration of the biological function of GC cells. We confirmed that the co‐culture of CAF promoted the viability (SNU‐1, *P* = 0.00832; SNU‐1+CAF, *P* = 0.0217; Figure 4a), invasion (SNU‐1, *P* = 0.00309; SNU‐1+CAF, *P* = 0.00168; Figure 4d) and migration of SNU‐1 cells (SNU‐1, *P* = 0.00326; SNU‐1+CAF, *P* = 0.0163; Figure 4c) while the apoptotic cell rate was reduced (SNU‐1, *P* = 0.00637; SNU‐1+CAF, *P* = 0.0296; Figure 4b); besides, the aggravated effect of CAF on GC cells was attenuated by the transfection of si‐PDPNs (SNU‐1‐si‐PDPNs + CAF: *P* = 0.0331, Figure 4a; *P* = 0.00709, Figure 4b; *P* = 0.00805, Figure 4c; *P* = 0.00261, Figure 4d). Previous studies indicated that the interaction between tumour cells and CAFs was achieved in a paracrine manner depending on secreted proteins and cytokines (Nallasamy et al., 2021). Therefore, we inferred that PDPN, as a secreted protein, participated in an interaction with the TME. Our results showed that the supplementation of exogenous PDPN reversed the effects of si‐PDPNs in a CAF co‐culture system (si‐PDPNs + rPDPN +CAF: *P* = 0.0273, Figure 4a; *P* = 0.0354, Figure 4b; *P* = 0.0394, Figure 4c; *P* = 0.0109, Figure 4d). These findings suggested that PDPN was closely associated with CAF‐induced GC progression.

### 3.5 PDPN contributed to the activation of CAF in a tumour microenvironment

**FIGURE 5:**
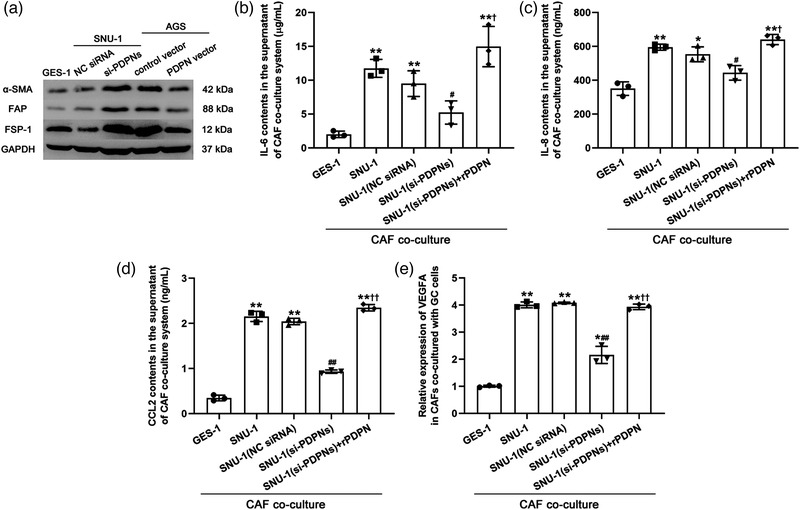
PDPN alteration in GC cells contributed to the activation of CAF in tumour microenvironment. (a) The protein expression of α‐SMA, FAP and FSP‐1 in CAF co‐cultured with SNU‐1 cells. (b–d) The contents of IL‐6 (b), IL‐8 (c), and CCL2 (d) in the supernatant of CAF after co‐cultured with SNU‐1 cells. (e) The relative mRNA expression of VEGFA in in CAF after co‐culture with SNU‐1 cells or si‐RNA‐transfected SNU‐1 cells. ^*^ *P* < 0.05 and ^**^ *P* < 0.01 versus GES‐1 cell line; ^#^ *P* < 0.05 and ^##^ *P* < 0.01 versus SNU‐1 cell line; ^†^ *P* < 0.05 and ^††^ *P* < 0.01 versus SNU‐1 (si‐PDPNs) cell line. All data are presented as mean ± SD (*n* = 3).

We further determined whether the expression of PDPN in GC cells promotes the activation of CAFs in the TME. The protein expression of CAF‐related biomarker genes, smooth muscle α‐actin (α‐SMA), fibroblast‐associated protein (FAP) and fibroblast‐specific protein 1 (FSP‐1), was markedly activated in GC cell‐co‐cultured CAFs and regulated by the expression of PDPN in SNU‐1 cells (Figure 5a). Subsequently, we confirmed that the contents of CAF‐secreted cytokines, IL‐6 (SNU‐1, *P* = 0.00180; Figure 5b), IL‐8 (SNU‐1, *P* = 0.00116; Figure 5c) and CCL2 (SNU‐1, *P* = 0.00617; Figure 5d), and the expression of VEGFA (SNU‐1, *P* = 0.00164; Figure 5e) in the GC‐cultured system were dramatically increased; these effects were inhibited in the system co‐cultured with PDPN‐silenced SNU‐1 cells (si‐PDPNs: *P* = 0.0205, Figure 5b; *P* = 0.0181, Figure 5c; *P* = 0.00503, Figure 5d; *P* = 0.00283, Figure 5e), which was reversed by the exogenous supplementation of PDPN (si‐PDPNs + rPDPN: *P* = 0.0170, Figure 5b; *P* = 0.0201, Figure 5c; *P* = 0.00914, Figure 5d; *P* = 0.00219, Figure 5e).

## 4 DISCUSSION

Changes in PDPN had been reported in different human cancers. PDPN upregulation potentiated invasion of low invasive oral squamous cell carcinoma cells by increasing the formation of invadopodia and the degradation of extracellular matrix (Hwang et al., 2012). Abnormal expression of PDPN was also involved in the invasion and metastasis of head and neck squamous cell carcinoma (Sun et al., 2013). It was also observed that PDPN was increased in sinonasal squamous cell carcinoma and clear cell renal cell carcinoma and associated with low rates of patients’ overall survival and disease‐free survival (Wang et al., 2020; Xia et al., 2016). These studies raised the possibility that PDPN plays an important role in regulating the biological function of cancer cells. Besides, Hu et al. (2020) suggested that the high expression of PDPN in GC was highly associated with macrophage, dendritic cell and T cell infiltration. Our findings are consistent with the previous studies and illustrate that PDPN is increased in GC tissues and cell lines. High PDPN expression level in patients with GC was linked to their poor survival outcome. Furthermore, PDPN was also confirmed to foster cell viability, migration, invasion and suppressed apoptosis of GC cells.

In this research, we observed that the knockdown of PDPN dramatically depressed the activation of ezrin in GC cells, which was shown to be a contributor to cancer progression (Kong et al., 2016; Qureshi‐Baig et al., 2020). Ezrin, a member of the ezrin–radixin–moesin protein family, is strongly expressed in many types of cancers. The enhanced expression of ezrin promoted proliferation, invasion and EMT of ovarian cancer cells (Li et al., 2021) and contributed to aggressive tumour characteristics and poor prognosis of breast cancer (Li et al., 2019). Enhanced ezrin activation was linked with tumour grade, TNM stage and lymph node metastasis in GC (Liang et al., 2017). Besides, Suzuki et al. (2015) suggested that ezrin exerted a vital role in the invasion of lung cancer within PDPN‐expressing CAF‐composed TME. To some extent, these studies provide support for our conclusions. In addition, it was reported that ROCK could invoke the recruitment and generation of CAFs to maintain breast cancer phenotype (Boyle et al., 2020). Our study indicated that ROCK reversed the action of PDPN knockdown to promote the activation of CAFs and the release of inflammatory factors to accelerate GC progression, while NSC668394 had the same inhibitory effects as PDPN knockdown.

CAFs are the most abundant stromal cells in the TME and interact with cancer cells to aggravate their malignant behaviours. Al‐Kharashi et al. (2021) suggested that targeting CAFs or decreasing the carcinogenic effect of CAFs could be effective for suppressing cancer progression. A recent study confirmed that driving the interaction between tumour cells and CAFs facilitated the malignant progression of tumours (Chen et al., 2021). Consistently, we found that the expression of PDPN in GC cells activated CAFs and promoted the secretion of tumour‐promoting cytokines in CAFs. Interestingly, several studies demonstrated that cancer cells and PDPN‐expressing CAFs might contribute to a malignant microenvironment for tumour tissues (Hoshino et al., 2011). Yurugi et al. (2017) considered that the PDPN expression in CAFs was a marker of poor prognosis in patients with lung squamous cell carcinoma. A previous study pointed out that PDPN‐expressing CAFs facilitated the development of invasive ductal carcinoma (Shindo et al., 2013). However, the role of PDPN in the interaction between CAFs and GC cells is still vague and needs further exploration.

## 5 Conclusion

In conclusion, PDPN was upregulated in GC tissues and cells and negatively associated with the survival outcome of GC patients. Besides, PDPN promoted GC cell viability, invasion and migration by activating ezrin, while cell apoptosis was inhibited. Importantly, PDPN activated CAFs and contributed to the interaction between GC cells and CAFs to induce the malignant biological behaviours of GC cells. These interesting findings might provide a foundation for GC therapies.

## AUTHOR CONTRIBUTIONS

Ying Song designed the study. Yueli Tian, Xin Chen and Xiaodong Wang performed the experiments. Yueli Tian wrote this manuscript and Ying Song oversaw language editing. All authors have read and approved the final version of this manuscript and agree to be accountable for all aspects of the work in ensuring that questions related to the accuracy or integrity of any part of the work are appropriately investigated and resolved. All persons designated as authors qualify for authorship, and all those who qualify for authorship are listed. The authors participating in this study all provided written informed consents for the publication.

## CONFLICT OF INTEREST

The authors in this study declared no competing interests.

## Supporting information

Statistical Summary Document

Figures S1–S3

## Data Availability

Data from this study was available from the corresponding author for appropriate reasons.
