# Supplementary material for: Podoplanin promotes the carcinogenicity of gastric cancer by activating ezrin and mediating the crosstalk between tumour cells and cancer‐associated fibroblasts
Source: Exp Physiol. 2023 Mar 28;108(5):740–51. doi: 10.1113/EP090172 (PMC10988511; doi:10.1113/EP090172)
Supplement: Supplementary file 1 — Statistical Summary Document [file EPH-108-740-s001.docx]

**Manuscript Title:** PDPN promotes the carcinogenicity of gastric cancer via activating Ezrin and mediating the crosstalk between tumor cells and cancer-associated fibroblasts

**Authors:**

**Animal model used, if applicable:** Not applicable.

**Underlying hypothesis:** This investigation tests the hypothesis that PDPN mediates gastric cancer progression via Ezrin activation and participants in the interaction with cancer-associated fibroblasts in tumor microenvironment.

**Definitions of ‘n’:**

Question 1: n = individual number of tissue samples.

Question 2: n = number of subjects.

Question 3~Question 21: n = number of experimental repeats of every group.

**Statistical summary table:**

| Experimental question number* | Finding/ conclusion | Experimental location/ variable  e.g. muscle, neocortex or genotype | Mean value  (or other summary statistic) | SD | n val. | P** | Units | Data comparisons  e.g. WT vs KO | Statistical test | Any other variable  e.g. subjects’ age or sex | Figure/ table in which data are presented | Comments  e.g. observation |
| --- | --- | --- | --- | --- | --- | --- | --- | --- | --- | --- | --- | --- |
| 1. PDPN mRNA expression relative to normal | Expression level of PDPN is higher in tumor tissues than normal tissues | Normal tissues | 1.00 | 0.366 | 35 | - | Fold | Normal tissues vs tumor tissues | Students’ t test, unpaired | nA | 1A | nA |
|  |  | Tumor tissues | 2.39 | 0.775 | 35 | **<0.0001** | Fold |  |  | nA | 1A | nA |
| 2. overall survival | Low-expression of PDPN group has longer survival time than high-expression group | High PDPN expression | 27.00 | nA | 20 | **-** | Month | High PDPN expression vs Low PDPN expression | Kaplan-Meier | nA | 1E | nA |
|  |  | Low PDPN expression | 45.00 | nA | 15 | **0.03013** | month |  |  | nA | 1E | nA |
| 3. PDPN mRNA expression relative to GES-1 | PDPN expression in AGS, HGC-27, MKN-45, SNU-1, Hs746T are higher than GES-1 | GES-1 | 1.00 | 0.102 | 3 | - | Fold | - | Students’ t test, unpaired | nA | 1F | nA |
|  |  | AGS | 2.24 | 0.398 | 3 | **0.00651** | Fold | AGS vs GES-1 |  | nA | 1F | nA |
|  |  | HGC-27 | 1.83 | 0.0519 | 3 | 0.0136 | Fold | HGC-27 vs GES-1 |  | nA | 1F | nA |
|  |  | MKN-45 | 3.20 | 0.267 | 3 | 0.00361 | Fold | MKN-45 vs GES-1 |  | nA | 1F | nA |
|  |  | SNU-1 | 3.71 | 0.661 | 3 | **0.00821** | Fold | SNU-1 vs GES-1 |  | nA | 1F | nA |
|  |  | Hs.746T | 2.29 | 0.0469 | 3 | 0.00228 | fold | Hs.746T vs GES-1 |  | nA | 1F | nA |
| 4. PDPN mRNA expression relative to untreated group | PDPN expression in si-PDPNb and si-PDPNc are lower than untreated or NC siRNA groups | untreated | 1.00 | 0.115 | 3 | - | Fold | - | Students’ t test, unpaired | nA | 2A | nA |
|  |  | NC siRNA | 1.07 | 0.0438 | 3 | 0.451 | Fold | NC siRNA vs untreated |  | nA | 2A | nA |
|  |  | Si-PDPNa | 0.752 | 0.109 | 3 | 0.0236 | Fold | Si-PDPNa vs untreated |  | nA | 2A | nA |
|  |  | Si-PDPNb | 0.325 | 0.0527 | 3 | **0.00682** | Fold | Si-PDPNb vs untreated |  | nA | 2A | nA |
|  |  | Si-PDPNc | 0.412 | 0.0525 | 3 | **0.00703** | Fold | Si-PDPNb vs untreated |  | nA | 2A | nA |
| 5. PDPN mRNA expression relative to untreated group | PDPN expression in 5 µg PDPN vector is higher than untreated or control vector groups | Untreated | 1.00 | 0.0363 | 3 | - | Fold | - | Students’ t test, unpaired | nA | 2C | nA |
|  |  | Control vector | 0.958 | 0.0291 | 3 | 0.531 | Fold | Control vector vs untreated |  | nA | 2C | nA |
|  |  | 1 µg PDPN vector | 1.06 | 0.0731 | 3 | 0.433 | Fold | 1 µg PDPN vector vs untreated |  | nA | 2C | nA |
|  |  | 2 µg PDPN vector | 2.02 | 0.314 | 3 | 0.0261 | Fold | 2 µg PDPN vector vs untreated |  | nA | 2C | nA |
|  |  | 5 µg PDPN vector | 3.20 | 0.139 | 3 | **0.0137** | Fold | 5 µg PDPN vector vs untreated |  | nA | 2C | nA |
| 6. OD values relative to NC siRNA SNU-1 or control vector AGS | OD values of si-PDPNs are lower than NC siRNA | 24 h NC siRNA SNU-1 | 1.00 | 0.0468 | 33 | - | Fold | - | Students’ t test, unpaired | nA | 2E | nA |
|  |  | 48 h NC siRNA SNU-1 | 2.18 | 0.172 | 3 | - | Fold | - |  | nA | 2E | nA |
|  |  | 72 h NC siRNA SNU-1 | 4.24 | 0.274 | 3 | - | Fold | - |  | nA | 2E | nA |
|  |  | 96 h NC siRNA SNU-1 | 7.38 | 0.336 | 3 | - | Fold | - |  | nA | 2E | nA |
|  |  | 24 h si-PDPNs SNU-1 | 1.01 | 0.0417 | 3 | 0.712 | Fold | 24 h si-PDPNs vs 24 h NC siRNA |  | nA | 2E | nA |
|  |  | 48 h si-PDPNs SNU-1 | 1.72 | 0.0404 | 3 | 0.0395 | Fold | 48 h si-PDPNs vs 48 h NC siRNA |  | nA | 2E | nA |
|  |  | 72 h si-PDPNs SNU-1 | 2.96 | 0.117 | 3 | **0.00815** | Fold | 72 h si-PDPNs vs 72 h NC siRNA |  | nA | 2E | nA |
|  |  | 96 h si-PDPNs SNU-1 | 4.13 | 0.147 | 3 | **0.00519** | Fold | 96 h si-PDPNs vs 96 h NC siRNA |  | nA | 2E | nA |
|  | OD values of PDPN vector are higher than control vector | 24 h control vector AGS | 1.00 | 0.0282 | 3 | - | Fold | - |  | nA | 2E | nA |
|  |  | 48 h control vector AGS | 1.60 | 0.136 | 3 | - | Fold | - |  | nA | 2E | nA |
|  |  | 72 h control vector AGS | 2.53 | 0.0364 | 3 | - | Fold | - |  | nA | 2E | nA |
|  |  | 96 h control vector AGS | 4.19 | 0.172 | 3 | - | Fold | - |  | nA | 2E | nA |
|  |  | 24 h PDPN vector AGS | 0.999 | 0.0591 | 3 | 0.661 | Fold | 24 h PDPN vector vs 24 h control vector |  | nA | 2E | nA |
|  |  | 48 h PDPN vector AGS | 1.93 | 0.0977 | 3 | 0.193 | Fold | 48 h PDPN vector vs 48 h control vector |  | nA | 2E | nA |
|  |  | 72 h PDPN vector AGS | 3.79 | 0.111 | 3 | **0.00694** | Fold | 72 h PDPN vector vs 72 h control vector |  | nA | 2E | nA |
|  |  | 96 h PDPN vector AGS | 6.15 | 0.186 | 3 | **0.00138** | Fold | 96 h PDPN vector vs 96 h control vector |  | nA | 2E | nA |
| 7. cell apoptosis rate | Si-PDPNs cell apoptosis rate is higher than NC siRNA | NC siRNA SNU-1 | 5.38 | 0.554 | 3 | - | % | - | Students’ t test, unpaired | nA | 2F | nA |
|  |  | Si-PDPNs SNU-1 | 19.7 | 2.38 | 3 | **0.00837** | % | Si-PDPNs vs NC siRNA |  | nA | 2F | nA |
|  | PDPN vector cell apoptosis rate is lower than control vector | Control vector AGS | 6.74 | 0.164 | 3 | - | % | - |  | nA | 2F | nA |
|  |  | PDPN vector AGS | 14.7 | 0.810 | 3 | **0.00604** | % | PDPN vector vs control vector |  | nA | 2F | nA |
| 8. wound closure rate | Si-PDPNs wound closure is weaker than NC siRNA | NC siRNA SNU-1 | 16.7 | 1.17 | 3 | - | % | - | Students’ t test, unpaired | nA | 2G | nA |
|  |  | Si-PDPNs SNU-1 | 4.06 | 0.465 | 3 | **0.00573** | % | Si-PDPNs vs NC siRNA |  | nA | 2G | nA |
|  | PDPN vector wound closure is stronger than control vector | Control vector AGS | 13.5 | 1.57 | 3 | - | % | - |  | nA | 2G | nA |
|  |  | PDPN vector AGS | 23.3 | 4.59 | 3 | **0.0328** | % | PDPN vector vs control vector |  | nA | 2G | nA |
| 9. number of invaded cells | Si-PDPNs invasion number is less than NC siRNA | NC siRNA SNU-1 | 68.6 | 7.83 | 3 | - | cell | - | Students’ t test, unpaired | nA | 2H | nA |
|  |  | Si-PDPNs SNU-1 | 27.7 | 1.93 | 3 | **0.00731** | cell | Si-PDPNs vs NC siRNA |  | nA | 2H | nA |
|  | PDPN vector invasion number is more than control vector | Control vector AGS | 128 | 11.7 | 3 | - | cell | - |  | nA | 2H | nA |
|  |  | PDPN vector AGS | 223 | 30.8 | 3 | **0.0173** | cell | PDPN vector vs control vector |  | nA | 2H | nA |
| 10. OD values relative to NC siRNA SNU-1 or control vector AGS | OD values of si-PDPNs are lower than NC siRNA; OD values of si-PDPNs+ROCK are higher than si-PDPNs | 24 h NC siRNA SNU-1 | 1.00 | 0.0664 | 3 | - | Fold | - | Students’ t test, unpaired | nA | 3B | nA |
|  |  | 48 h NC siRNA SNU-1 | 2.08 | 0.0301 | 3 | - | Fold | - |  | nA | 3B | nA |
|  |  | 72 h NC siRNA SNU-1 | 4.14 | 0.244 | 3 | **-** | Fold | - |  | nA | 3B | nA |
|  |  | 96 h NC siRNA SNU-1 | 6.99 | 0.0972 | 3 | **-** | Fold | - |  | nA | 3B | nA |
|  |  | 24 h si-PDPNs SNU-1 | 0.996 | 0.0310 | 3 | 0.823 | Fold | 24 h si-PDPNs vs 24 h NC siRNA |  | nA | 3B | nA |
|  |  | 48 h si-PDPNs SNU-1 | 1.71 | 0.0697 | 3 | **0.0337** | Fold | 48 h si-PDPNs vs 48 h NC siRNA |  | nA | 3B | nA |
|  |  | 72 h si-PDPNs SNU-1 | 2.66 | 0.0603 | 3 | **0.00258** | Fold | 72 h si-PDPNs vs 72 h NC siRNA |  | nA | 3B | nA |
|  |  | 96 h si-PDPNs SNU-1 | 3.80 | 0.134 | 3 | **0.00129** | Fold | 96 h si-PDPNs vs 96 h NC siRNA |  | nA | 3B | nA |
|  |  | 24 h si-PDPNs+ROCK SNU-1 | 1.00 | 0.0781 | 3 | 0.964 | Fold | 24 h si-PDPNs+ROCK vs 24 h si-PDPNs |  | nA | 3B | nA |
|  |  | 48 h si-PDPNs+ROCK SNU-1 | 2.02 | 0.136 | 3 | 0.0931 | Fold | 48 h si-PDPNs+ROCK vs 48 h si-PDPNs |  | nA | 3B | nA |
|  |  | 72 h si-PDPNs+ROCK SNU-1 | 3.82 | 0.227 | 3 | **0.00635** | Fold | 72 h si-PDPNs+ROCK vs 72 h si-PDPNs |  | nA | 3B | nA |
|  |  | 96 h si-PDPNs+ROCK SNU-1 | 6.01 | 0.191 | 3 | **0.00594** | Fold | 96 h si-PDPNs+ROCK vs 96 h si-PDPNs |  | nA | 3B | nA |
|  | OD values of PDPN vector are higher than control vector; OD values of PDPN vector+NSC668394 are higher than PDPN vector | 24 h control vector AGS | 1.00 | 0.0337 | 3 | - | Fold | - |  | nA | 3C | nA |
|  |  | 48 h control vector AGS | 1.65 | 0.0424 | 3 | - | Fold | - |  | nA | 3C | nA |
|  |  | 72 h control vector AGS | 2.56 | 0.0769 | 3 | - | Fold | - |  | nA | 3C | nA |
|  |  | 96 h control vector AGS | 4.41 | 0.0896 | 3 | - | Fold | - |  | nA | 3C | nA |
|  |  | 24 h PDPN vector AGS | 1.01 | 0.0743 | 3 | 0.863 | Fold | 24 h PDPN vector vs 24 h control vector |  | nA | 3C | nA |
|  |  | 48 h PDPN vector AGS | 2.09 | 0.0392 | 3 | **0.0435** | Fold | 48 h PDPN vector vs 48 h control vector |  | nA | 3C | nA |
|  |  | 72 h PDPN vector AGS | 4.11 | 0.124 | 3 | **0.00863** | Fold | 72 h PDPN vector vs 72 h control vector |  | nA | 3C | nA |
|  |  | 96 h PDPN vector AGS | 6.00 | 0.141 | 3 | **0.00571** | Fold | 96 h PDPN vector vs 96 h control vector |  | nA | 3C | nA |
|  |  | 24 h PDPN vector+NSC668394 AGS | 0.999 | 0.0515 | 3 | 0.762 | Fold | 24 h PDPN vector+NSC668394 vs 24 h PDPN vector |  | nA | 3C | nA |
|  |  | 48 h PDPN vector+NSC668394 AGS | 1.63 | 0.0345 | 3 | **0.0433** | Fold | 48 h PDPN vector+NSC668394 vs 48 h PDPN vector |  | nA | 3C | nA |
|  |  | 72 h PDPN vector+NSC668394 AGS | 2.81 | 0.0768 | 3 | **0.00912** | Fold | 72 h PDPN vector+NSC668394 vs 72 h PDPN vector |  | nA | 3C | nA |
|  |  | 96 h PDPN vector+NSC668394 AGS | 3.90 | 0.178 | 3 | **0.00662** | Fold | 96 h PDPN vector+NSC668394 vs 96 h PDPN vector |  | nA | 3C | nA |
| 11. cell apoptosis rate | Si-PDPNs cell apoptosis rate is higher than NC siRNA; Si-PDPNs+ROCK cell apoptosis rate is lower than si-PDPNs | NC siRNA SNU-1 | 5.38 | 0.554 | 3 | - | % | - | Students’ t test, unpaired | nA | 3D | nA |
|  |  | Si-PDPNs SNU-1 | 19.75 | 2.38 | 3 | **0.00815** | % | Si-PDPNs vs NC siRNA |  | nA | 3D | nA |
|  |  | si-PDPNs+ROCK SNU-1 | 8.14 | 0.534 | 3 | **0.00631** | % | si-PDPNs+ROCK vs Si-PDPNs |  | nA | 3D | nA |
|  | PDPN vector cell apoptosis rate is lower than control vector; PDPN vector+NSC668394 cell apoptosis rate is higher than PDPN vector | Control vector AGS | 14.7 | 0.810 | 3 | - | % | - |  | nA | 3D | nA |
|  |  | PDPN vector AGS | 6.74 | 0.164 | 3 | **0.00791** | % | PDPN vector vs control vector |  | nA | 3D | nA |
|  |  | PDPN vector+NSC668394 AGS | 16.2 | 0.696 | 3 | **0.0132** | % | PDPN vector+NSC668394 vs PDPN vector |  | nA | 3D | nA |
| 12. wound closure rate | Si-PDPNs wound closure is weaker than NC siRNA; Si-PDPNs+ROCK wound closure is stronger than si-PDPNs | NC siRNA SNU-1 | 16.7 | 1.17 | 3 | - | % | - | Students’ t test, unpaired | nA | 3E | nA |
|  |  | Si-PDPNs SNU-1 | 4.06 | 0.465 | 3 | **0.00362** | % | Si-PDPNs vs NC siRNA |  | nA | 3E | nA |
|  |  | si-PDPNs+ROCK SNU-1 | 13.1 | 1.38 | 3 | **0.00518** | % | si-PDPNs+ROCK vs Si-PDPNs |  | nA | 3E | nA |
|  | PDPN vector wound closure is stronger than control vector; PDPN vector+NSC668394 wound closure is weaker than PDPN vector | Control vector AGS | 13.5 | 1.57 | 3 | - | % | - |  | nA | 3E | nA |
|  |  | PDPN vector AGS | 23.3 | 4.59 | 3 | **0.0196** | % | PDPN vector vs control vector |  | nA | 3E | nA |
|  |  | PDPN vector+NSC668394 AGS | 15.3 | 2.36 | 3 | **0.0381** | % | PDPN vector+NSC668394 vs PDPN vector |  | nA | 3E | nA |
| 13. number of invaded cells | Si-PDPNs invasion number is less than NC siRNA; Si-PDPNs+ROCK invasion number is more than Si-PDPNs | NC siRNA SNU-1 | 68.6 | 7.83 | 3 | - | cell | - | Students’ t test, unpaired | nA | 3F | nA |
|  |  | Si-PDPNs SNU-1 | 27.7 | 1.93 | 3 | **0.0364** | cell | Si-PDPNs vs NC siRNA |  | nA | 3F | nA |
|  |  | si-PDPNs+ROCK SNU-1 | 52.8 | 10.0 | 3 | **0.0173** | cell | si-PDPNs+ROCK vs Si-PDPNs |  | nA | 3F | nA |
|  | PDPN vector invasion number is more than control vector; PDPN vector+NSC668394 invasion number is less than PDPN vector | Control vector AGS | 128 | 11.7 | 3 | - | cell | - |  | nA | 3F | nA |
|  |  | PDPN vector AGS | 223 | 30.8 | 3 | **0.0261** | cell | PDPN vector vs control vector |  | nA | 3F | nA |
|  |  | PDPN vector+NSC668394 AGS | 162 | 21.3 | 3 | **0.0165** | cell | PDPN vector+NSC668394 vs PDPN vector |  | nA | 3F | nA |
| 14. OD values relative to NC siRNA SNU-1 or control vector AGS | OD value of SNU-1 is higher than GES-1; OD value of SNU-1+CAF is higher than SNU-1; OD value of SNU-1(si-PDPNs)+CAF is lower than SNU-1+CAF; OD value of SNU-1(si-PDPNs)+rPDPN+CAF is higher than SNU-1(si-PDPNs)+CAF | GES-1 | 3.14 | 0.296 | 3 | - | Fold | - | Students’ t test, unpaired | nA | 4A | nA |
|  |  | SNU-1 | 6.98 | 0.998 | 3 | **0.00832** | Fold | SNU-1 vs GES-1 |  | nA | 4A | nA |
|  |  | SNU-1+CAF | 10.4 | 2.26 | 3 | **0.0217** | Fold | SNU-1+CAF vs SNU-1 |  | nA | 4A | nA |
|  |  | SNU-1(si-PDPNs)+CAF | 6.38 | 0.634 | 3 | **0.0331** | Fold | SNU-1(si-PDPNs)+CAF vs SNU-1+CAF |  | nA | 4A | nA |
|  |  | SNU-1(si-PDPNs)+rPDPN+CAF | 8.79 | 1.43 | 3 | **0.0273** | Fold | SNU-1(si-PDPNs)+rPDPN+CAF vs SNU-1(si-PDPNs)+CAF |  | nA | 4A | nA |
| 15. cell apoptosis rate | Cell apoptosis rate of SNU-1 is lower than GES-1; Cell apoptosis rate of SNU-1+CAF is lower than SNU-1; Cell apoptosis rate of SNU-1(si-PDPNs)+CAF is higher than SNU-1+CAF; Cell apoptosis rate of SNU-1(si-PDPNs)+rPDPN+CAF is lower than SNU-1(si-PDPNs)+CAF | GES-1 | 8.04 | 0.714 | 3 | - | % | - | Students’ t test, unpaired | nA | 4B | nA |
|  |  | SNU-1 | 5.38 | 0.554 | 3 | **0.00637** | % | SNU-1 vs GES-1 |  | nA | 4B | nA |
|  |  | SNU-1+CAF | 3.96 | 0.337 | 3 | **0.0296** | % | SNU-1+CAF vs SNU-1 |  | nA | 4B | nA |
|  |  | SNU-1(si-PDPNs)+CAF | 16.3 | 1.22 | 3 | **0.00709** | % | SNU-1(si-PDPNs)+CAF vs SNU-1+CAF |  | nA | 4B | nA |
|  |  | SNU-1(si-PDPNs)+rPDPN+CAF | 11.7 | 1.13 | 3 | **0.0354** | % | SNU-1(si-PDPNs)+rPDPN+CAF vs SNU-1(si-PDPNs)+CAF |  | nA | 4B | nA |
| 16. wound closure rate | SNU-1 wound closure is stronger than GES-1; SNU-1+CAF wound closure is stronger than SNU-1; SNU-1(si-PDPNs)+CAF wound closure is weaker than SNU-1+CAF; SNU-1(si-PDPNs)+rPDPN+CAF wound closure is stronger than SNU-1(si-PDPNs)+CAF | GES-1 | 3.25 | 0.624 | 3 | - | % | - | Students’ t test, unpaired | nA | 4C | nA |
|  |  | SNU-1 | 16.7 | 1.17 | 3 | **0.00326** | % | SNU-1 vs GES-1 |  | nA | 4C | nA |
|  |  | SNU-1+CAF | 26.3 | 1.92 | 3 | **0.0163** | % | SNU-1+CAF vs SNU-1 |  | nA | 4C | nA |
|  |  | SNU-1(si-PDPNs)+CAF | 14.8 | 2.78 | 3 | **0.00805** | % | SNU-1(si-PDPNs)+CAF vs SNU-1+CAF |  | nA | 4C | nA |
|  |  | SNU-1(si-PDPNs)+rPDPN+CAF | 19.8 | 3.41 | 3 | **0.0394** | % | SNU-1(si-PDPNs)+rPDPN+CAF vs SNU-1(si-PDPNs)+CAF |  | nA | 4C | nA |
| 17. number of invaded cells | SNU-1 invasion number is more than GES-1; SNU-1+CAF invasion number is more than SNU-1; SNU-1(si-PDPNs)+CAF invasion number is less than SNU-1+CAF; SNU-1(si-PDPNs)+rPDPN+CAF invasion number is more than SNU-1(si-PDPNs)+CAF | GES-1 | 23.3 | 7.21 | 3 | - | cell | - | Students’ t test, unpaired | nA | 4D | nA |
|  |  | SNU-1 | 68.6 | 7.82 | 3 | **0.00309** | cell | SNU-1 vs GES-1 |  | nA | 4D | nA |
|  |  | SNU-1+CAF | 117 | 15.5 | 3 | **0.00168** | cell | SNU-1+CAF vs SNU-1 |  | nA | 4D | nA |
|  |  | SNU-1(si-PDPNs)+CAF | 74.6 | 6.69 | 3 | **0.00261** | cell | SNU-1(si-PDPNs)+CAF vs SNU-1+CAF |  | nA | 4D | nA |
|  |  | SNU-1(si-PDPNs)+rPDPN+CAF | 96.9 | 7.36 | 3 | **0.0109** | cell | SNU-1(si-PDPNs)+rPDPN+CAF vs SNU-1(si-PDPNs)+CAF |  | nA | 4D | nA |
| 18. IL-6 contents | IL-6 content of SNU-1 is higher than GES-1; SNU-1(si-PDPNs) is lower than SNU-1(NC siRNA); SNU-1(si-PDPNs)+rPDPN is higher than SNU-1(si-PDPNs) | GES-1 | 2.00 | 0.385 | 3 | - | µg/mL | - | Students’ t test, unpaired | nA | 5B | nA |
|  |  | SNU-1 | 11.7 | 1.08 | 3 | **0.00180** | µg/mL | SNU-1 vs GES-1 |  | nA | 5B | nA |
|  |  | SNU-1(NC siRNA) | 9.49 | 1.55 | 3 | 0.806 | µg/mL | SNU-1(NC siRNA) vs SNU-1 |  | nA | 5B | nA |
|  |  | SNU-1(si-PDPNs) | 5.23 | 1.40 | 3 | **0.0205** | µg/mL | SNU-1(si-PDPNs) vs SNU-1(NC siRNA) |  | nA | 5B | nA |
|  |  | SNU-1(si-PDPNs)+rPDPN | 15.0 | 2.43 | 3 | **0.0170** | µg/mL | SNU-1(si-PDPNs)+rPDPN vs SNU-1(si-PDPNs) |  | nA | 5B | nA |
| 19. IL-8 contents | IL-8 content of SNU-1 is higher than GES-1; SNU-1(si-PDPNs) is lower than SNU-1(NC siRNA); SNU-1(si-PDPNs)+rPDPN is higher than SNU-1(si-PDPNs) | GES-1 | 350 | 32.8 | 3 | - | ng/mL | - | Students’ t test, unpaired | nA | 5C | nA |
|  |  | SNU-1 | 595 | 14.7 | 3 | **0.00116** | ng/mL | SNU-1 vs GES-1 |  | nA | 5C | nA |
|  |  | SNU-1(NC siRNA) | 553 | 35.8 | 3 | 0.923 | ng/mL | SNU-1(NC siRNA) vs SNU-1 |  | nA | 5C | nA |
|  |  | SNU-1(si-PDPNs) | 443 | 34.9 | 3 | **0.0181** | ng/mL | SNU-1(si-PDPNs) vs SNU-1(NC siRNA) |  | nA | 5C | nA |
|  |  | SNU-1(si-PDPNs)+rPDPN | 640 | 24.5 | 3 | **0.0201** | ng/mL | SNU-1(si-PDPNs)+rPDPN vs SNU-1(si-PDPNs) |  | nA | 5C | nA |
| 20. CCL2 contents | CCL2 content of SNU-1 is higher than GES-1; SNU-1(si-PDPNs) is lower than SNU-1(NC siRNA); SNU-1(si-PDPNs)+rPDPN is higher than SNU-1(si-PDPNs) | GES-1 | 0.346 | 0.0507 | 3 | - | ng/mL | - | Students’ t test, unpaired | nA | 5D | nA |
|  |  | SNU-1 | 2.15 | 0.0924 | 3 | **0.00617** | ng/mL | SNU-1 vs GES-1 |  | nA | 5D | nA |
|  |  | SNU-1(NC siRNA) | 2.04 | 0.0587 | 3 | 0.934 | ng/mL | SNU-1(NC siRNA) vs SNU-1 |  | nA | 5D | nA |
|  |  | SNU-1(si-PDPNs) | 0.93 | 0.0301 | 3 | **0.00503** | ng/mL | SNU-1(si-PDPNs) vs SNU-1(NC siRNA) |  | nA | 5D | nA |
|  |  | SNU-1(si-PDPNs)+rPDPN | 2.35 | 0.0578 | 3 | **0.00914** | ng/mL | SNU-1(si-PDPNs)+rPDPN vs SNU-1(si-PDPNs) |  | nA | 5D | nA |
| 21. VEGFA expression relative to GES-1 | VEGFA expression of SNU-1 is higher than GES-1; SNU-1(si-PDPNs) is lower than SNU-1(NC siRNA); SNU-1(si-PDPNs)+rPDPN is higher than SNU-1(si-PDPNs) | GES-1 | 1.00 | 0.0293 | 3 | - | Fold | - | Students’ t test, unpaired | nA | 5E | nA |
|  |  | SNU-1 | 4.01 | 0.0885 | 3 | **0.00164** | Fold | SNU-1 vs GES-1 |  | nA | 5E | nA |
|  |  | SNU-1(NC siRNA) | 4.09 | 0.0221 | 3 | 0.891 | Fold | SNU-1(NC siRNA) vs SNU-1 |  | nA | 5E | nA |
|  |  | SNU-1(si-PDPNs) | 2.16 | 0.258 | 3 | **0.00283** | Fold | SNU-1(si-PDPNs) vs SNU-1(NC siRNA) |  | nA | 5E | nA |
|  |  | SNU-1(si-PDPNs)+rPDPN | 3.94 | 0.0839 | 3 | **0.00219** | Fold | SNU-1(si-PDPNs)+rPDPN vs SNU-1(si-PDPNs) |  | nA | 5E | nA |

*You may use multiple lines for the same question to indicate multiple comparisons

** Authors may wish to make the text bold where p is considered significant against a stated confidence limit.
