# Supplementary figures and images for: Podoplanin promotes the carcinogenicity of gastric cancer by activating ezrin and mediating the crosstalk between tumour cells and cancer‐associated fibroblasts
Source: Exp Physiol. 2023 Mar 28;108(5):740–51. doi: 10.1113/EP090172 (PMC10988511; doi:10.1113/EP090172)

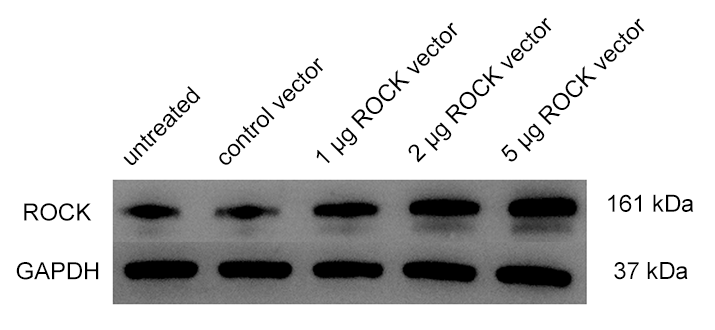

Supplement: Supplementary file 2 — Figures S1–S3 [file EPH-108-740-s002.zip › Figure S1.tif]

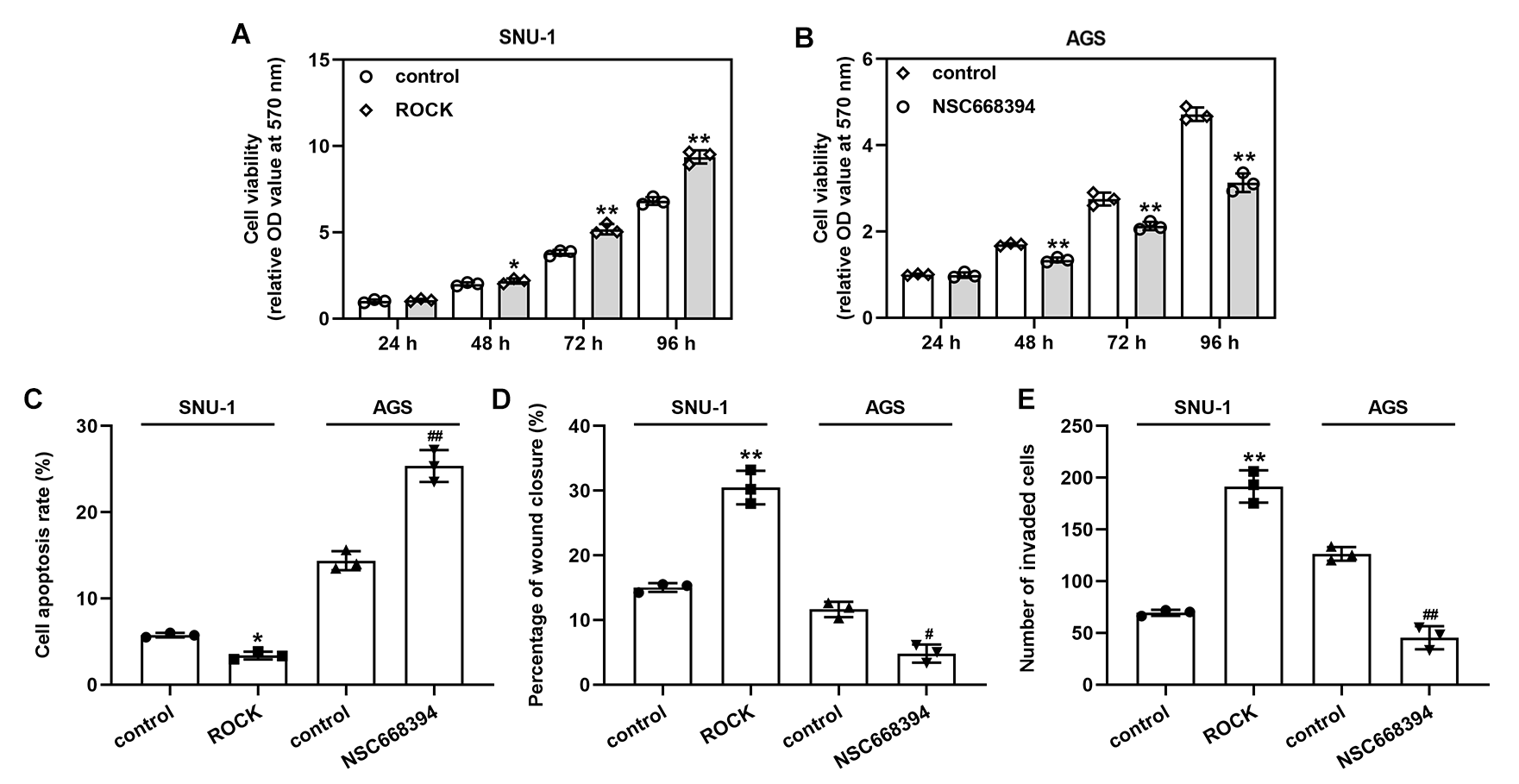

Supplement: Supplementary file 2 — Figures S1–S3 [file EPH-108-740-s002.zip › Figure S2.tif]

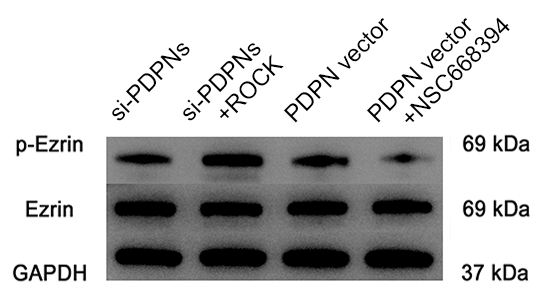

Supplement: Supplementary file 2 — Figures S1–S3 [file EPH-108-740-s002.zip › Figure S3.tif]
